# Beyond coverage: improving the quality of antenatal care delivery through integrated mentorship and quality improvement at health centers in rural Rwanda

**DOI:** 10.1186/s12913-018-2939-7

**Published:** 2018-02-23

**Authors:** Anatole Manzi, Laetitia Nyirazinyoye, Joseph Ntaganira, Hema Magge, Evariste Bigirimana, Leoncie Mukanzabikeshimana, Lisa R. Hirschhorn, Bethany Hedt-Gauthier

**Affiliations:** 1 Partners In Health, Kigali, Rwanda; 2 Partners In Health, Boston, USA; 3 College of Medicine and Health Sciences, University of Rwanda, Kigali, Rwanda; 4 Division of General Pediatrics, Boston Children’s Hospital, Boston, USA; 5 Institute for Healthcare Improvement, Addis Ababa, Ethiopia; 6 Division of Global Health Equity, Brigham and Women’s Hospital, Boston, USA; 7 Ministry of Health, Government of Rwanda, Kigali, Rwanda; 8 Northwestern University Feinberg School of Medicine, Chicago, USA; 9 Department of Global Health and Social Medicine, Harvard Medical School, Boston, USA

## Abstract

**Background:**

Inadequate antenatal care (ANC) can lead to missed diagnosis of danger signs or delayed referral to emergency obstetrical care, contributing to maternal mortality. In developing countries, ANC quality is often limited by skill and knowledge gaps of the health workforce. In 2011, the Mentorship, Enhanced Supervision for Healthcare and Quality Improvement (MESH-QI) program was implemented to strengthen providers’ ANC performance at 21 rural health centers in Rwanda. We evaluated the effect of MESH-QI on the completeness of danger sign assessments.

**Methods:**

Completeness of danger sign assessments was measured by expert nurse mentors using standardized observation checklists. Checklists completed from October 2010 to May 2011 (*n* = 330) were used as baseline measurement and checklists completed between February and November 2012 (12–15 months after the start of MESH-QI implementation) were used for follow-up. We used a mixed-effects linear regression model to assess the effect of the MESH-QI intervention on the danger sign assessment score, controlling for potential confounders and the clustering of effect at the health center level.

**Results:**

Complete assessment of all danger signs improved from 2.1% at baseline to 84.2% after MESH-QI (*p* <  0.001). Similar improvements were found for 20 of 23 other essential ANC screening items. After controlling for potential confounders, the improvement in danger sign assessment score was significant. However, the effect of the MESH-QI was different by intervention district and type of observed ANC visit. In Southern Kayonza District, the increase in the danger sign assessment score was 6.28 (95% CI: 5.59, 6.98) for non-first ANC visits and 5.39 (95% CI: 4.62, 6.15) for first ANC visits. In Kirehe District, the increase in danger sign assessment score was 4.20 (95% CI: 3.59, 4.80) for non-first ANC visits and 3.30 (95% CI: 2.80, 3.81) for first ANC visits.

**Conclusion:**

Assessment of critical danger signs improved under MESH-QI, even when controlling for nurse-mentees’ education level and previous training in focused ANC. MESH-QI offers an approach to enhance quality of care after traditional training and may be an approach to support newer providers who have not yet attended content-focused courses.

## Background

With the introduction of the millennium development goals (MDGs) in 2000, maternal death has been a focus of clinical and public health interventions globally [1–3]. Despite numerous clinical and public health interventions, the highest maternal mortality is still reported in sub-Saharan Africa [4], where poor quality healthcare contributed to failure to reach the MDG5 goal to reduce maternal and child mortality by three-quarters by 2015 [5–7]. This inadequate decline of maternal mortality in developing countries [8] calls for improved coverage and quality in health care for pregnant women.

Antenatal care (ANC) was initiated in the twentieth century as a strategy to prevent or ensure early treatment of pregnancy complications through systematic assessments, women’s education on positive behaviors, gestational age assessment, screening for fetal development and early detection of mother and baby abnormalities [6, 9]. There is evidence that ANC has the potential to reduce maternal mortality especially in low resource settings [10–12]. However, the quality of ANC is often hindered by gaps in knowledge and skills of care providers [13–17]. A study comparing thirty-eight countries found gaps in the quality of antenatal care delivery, including limited danger sign assessment and poor provision of essential counseling messages [18].

In Rwanda and other developing countries, poor quality of care is often exacerbated by the lack of basic equipment and low performance of health care workers [19–21]. While over 80% of the burden of diseases is addressed by health center nurses [22], the Africa Health Workforce Observatory estimated that Rwanda has only 1 nurse per 1493 people [23]. Such a low density of skilled professionals affects the overall quality of care at health center level. Although more than half of maternal deaths could be averted by adequate assessments and management of danger signs during ANC visits [9, 24–26], innovative strategies are needed to improve core maternal health care delivery processes [27, 28].

### Focused antenatal care training and supervision

In 2002, the World Health Organization adopted focused antenatal care (FANC) as a proactive strategy to detect and address critical needs for the mother and fetal wellbeing [29]. The goal of FANC is to identify opportunities for education and prevention or early management of problems that could affect pregnancy outcomes. In contrast to traditional ANC, FANC targets the individualized needs rather than relying solely on the frequency of ANC visits.

In 2003, Rwanda launched the implementation of FANC [30]. Health center providers attended classroom-based trainings that include a comprehensive review of ANC screenings so that these providers could develop an individualized child birth plan with each pregnant woman [31–33].

In Rwanda, in addition to FANC training, routine supervision visits were implemented as a strategy to facilitate the implementation of FANC. The Rwandan Ministry of Health recommended monthly supervision visits from district hospital (DH)‘s maternal and child health supervisors to health center-based ANC providers. Despite FANC trainings and routine supervision visits in Rwanda, there remained inconsistent and incomplete danger sign assessments during ANC visits, as has been observed in other countries in the region [22, 34–40]. We hypothesized that ongoing mentorship could address this gap by converting ANC assessment and management knowledge and skills into practice.

Historically, Rwanda has three main education tracks for nurses and midwives including A2, A1, and A0. A2 level nurses and midwives are trained to the secondary school level and covers basic clinical subjects and specific area of nursing specialties [41]. Since 2006, the Ministry of Health stopped training and deploying A2 level nurses and midwives, deeming their skill sets not sufficient to deliver high quality care services. Therefore, the ongoing efforts to upgrade A2 to A1 or A0 level may take several years [42]. In the meantime, A2 level nurses remain the bulk of nursing care at health center, fulfilling three functions including health promotion, preventative services provision, and primary healthcare delivery [43, 44].

### The MESH-QI intervention

Partners In Health (PIH) in collaboration with the Rwandan Ministry of Health (MoH) implemented a clinical Mentorship, Enhanced Supervision for Healthcare and Quality Improvement (MESH-QI) Program to improve the quality of care and systems in rural health centers in Rwanda [45]. During health center visits, MESH-QI mentors delivered provider-centered support including side-by-side mentorship, bedside teaching and clinical case review to improve knowledge, skills and effective communication techniques. All ANC providers, regardless of their training background, received mentorship visits every four to six weeks. In addition to mentorship, health center providers were coached on quality improvement, using Plan-Do-Study-Act cycle methodology, to help providers address facility issues that affected quality of maternal healthcare delivery [45]. The MESH-QI package is provided by expert nurse mentors with extensive experience as providers in specific clinical areas. These mentors are MoH employees who were trained in coaching and provided with ongoing support from an experienced gynecologist obstetrician, expert midwife and PIH’s QI specialist.

Previous evaluations have demonstrated that the MESH-QI model improved assessments and diagnosis across a variety of clinical programs, including the Integrated Management of Childhood Illness (IMCI), Prenatal Care and Integrated Management of Adolescent and Adulthood Illness (IMAI) and HIV [45, 46]. A qualitative study found positive perceptions and acceptability of the MESH-QI model from the perspective of the mentors, health care workers and district hospital managers, building health workers’ confidence in clinical diagnosis and case management [47].

In this study, we assess the impact of the MESH-QI intervention on the completeness of ANC assessment items, with a focus on danger signs. To our knowledge, no studies have evaluated the effectiveness of provider and systems-focused mentoring interventions to improve the quality of ANC at health centers in rural, sub-Saharan Africa. Our study findings could inform policy makers, managers and ANC providers wishing to improve the quality of ANC through integration of mentorship-based interventions in similar settings.

## Methods

### Study design and setting

This cross-sectional, pre-post study assesses the effect of MESH-QI on the completeness of ANC assessment items in rural Rwanda. We include all 21 PIH-supported public health centers, 8 in Southern Kayonza District and 13 in Kirehe District, collectively serving over 500,000 people [48]. These health centers, which are managed by the Rwandan MoH, were generally staffed by A2-level nurses (education level equivalent to secondary/high school) [49, 50]. All nurses working in the ANC clinic were eligible for mentoring and observation.

### Data collection

Baseline measurements were completed by the expert nurse mentors from October 2010 to May 2011 (*n* = 330) prior to any mentoring intervention to understand the pre-intervention clinical care activities. The follow-up measurement was completed by the mentors during support visits from February to November 2012 (*n* = 292), 12–15 months after the start of the MESH-QI intervention. The mentor observation checklists were adapted from the standards described in the Rwandan national ANC screening tool used at all health centers [51]. This tool listed the essential ANC assessment items including medical history, screening for seven danger signs (headache, blurry vision, facial swelling, convulsions, bleeding, loss of fluid, and painful contractions), measurement of vital signs, assessment of fetal well-being, communication and counseling [52].

### Data analysis

Data were analyzed using Stata v12 (College Station, TX: StataCorp LP).We use frequencies and percents to describe the nurse-mentee and facility characteristics. For all assessment areas, we compared completeness of assessment at baseline and after MESH-QI using the Chi-squared test.

The unit of analysis was the clinical encounter. The outcome of this study was the danger sign assessment score calculated based on equal weighting of the completion of each of the seven key danger sign assessments (0 indicating no danger sign was assessed and 7 indicating that all seven danger signs were assessed). We used interaction terms to assess whether the intervention district, completion of FANC training, level of nurse-mentee’s education, or type ANC visit (first or non-first ANC visit) modified the effect of the MESH-QI intervention. The interaction term was included in the final model if the interaction term variable was significant at the α = 0.05 level in bivariate analyses. We performed a multivariable linear regression analysis to assess the effect of MESH-QI on the danger sign assessment score, controlling for the following potential confounders: district (Southern Kayonza/Kirehe), nurse-mentee’s education level, nurse-mentee’s FANC training and type of ANC visit under observation (first vs others). Because a nurse could lead multiple clinical encounters, we used a random effect to account for clustering among observed ANC consultations conducted by the same nurse.

## Results

Observations were completed on 330 ANC visits conducted by 45 different nurses at baseline and 292 visits conducted by 35 different nurses during the follow-up period (Table 1). The number of nurses who had received FANC training varied over time; at baseline, 20 (44%) out of the 45 nurses had been trained in FANC compared to 21 (60%) out of 35 during follow-up period. Forty-three nurses (96%) at baseline had an A2 (high school) education compared to 32 (91%) during follow-up period. The remaining nurses had A1 (two to three years of post-secondary education as defined by the Rwanda Education Council) education.

**Table 1:** Demographics, study population, and case-observation characteristics

	Baseline		Follow-up	
	#	%	#	%
Demographics				
Number of health facilities	21		21	
Number of nurses observed	45		35	
Number of observations	330		292	
Nurse characteristics				
District				
Southern Kayonza	18	40	8	23
Kirehe	27	60	27	77
FANC trained	20	44	21	60
A2 level of education^a^	43	96	32	91
Case-observation characteristics				
Average number of observed cases per health center	16		14	
Antenatal care visit				
First	159	48	93	32
Others	171	52	199	68
Nurse providers trained in FANC^c^	164	50	166	57
Nurse’s education level				
A2^a^	317	96	266	91
A1^b^	13	4	26	9

^a^A2 level is a high school (secondary) level as defined by Rwanda education council

^b^A1 is two to three years of post-secondary education as defined by Rwanda education council

^c^FANC: Focused antenatal care including a thorough individualized surveillance of the pregnant woman, systematic screening of conditions and diseases, detection and management of pregnancy-related complications, and provision of counseling, preventive measures and support plan essential for safe pregnancy and delivery

For each of the seven danger sign assessment items, there was a significant improvement in completion at follow-up compared to baseline (*p* <  0.001) (Table 2). Overall the improvement in women with all danger signs assessed significantly improved, from 2.1% at baseline to 84.0% at follow-up (*p* <  0.001). Significant improvements were also found across other ANC assessment items. Observed ANC visits where nurses checked all vital signs and fetal wellbeing assessment items (fundal height, heart rate, movement, and position) improved significantly (1% to 55%, 37% to 89%, respectively, *p* <  0.001). Completeness of counseling improved significantly as well (2.2% to 51.0%, *p* <  0.001). Medical history assessment including previous surgeries, current medications, use of traditional medications, tobacco, and alcohol, domestic violence, and checking and documenting HIV status had less improvement, although the change was significant (2.1% to 14.0%, *p* <  0.001). No significant improvement was seen in proportion of observed cases assessed for previous surgery (28% to 29%, *p* = 0.796). The assessment of fetal heart rate remained high at both baseline and follow-up period (98% to 97%, *p* = 0.914).

**Table 2:** Completeness of antenatal care assessments before and after MESH-QI intervention

	Baseline		Follow-up		*P*-value
	n	%	n	%	
Danger signs					
Headache	79	24.0	278	95.2	< 0.001
Blurry vision	77	23.3	278	95.2	< 0.001
Facial swelling	184	56.0	290	99.3	< 0.001
Convulsions	57	17.3	275	94.1	< 0.001
Bleeding	134	41.0	285	98.0	< 0.001
Loss of fluid	76	23.0	267	91.4	< 0.001
Painful contractions	91	28.0	264	90.4	< 0.001
Composite	7	2.1	246	84.2	< 0.001
Medical history					
Previous surgeries	92	28.0	85	29.0	0.734
Current medications	11	3.3	41	14.0	< 0.001
Traditional medications/herbs	7	2.1	41	14.0	< 0.001
Tobacco use	8	2.4	38	13.1	< 0.001
Alcohol	10	3.0	39	13.5	< 0.001
Domestic violence	17	5.2	36	12.5	0.001
HIV status checked and documented	66	42.0	80	86.0	< 0.001
Composite	7	2.1	40	14.0	< 0.001
Vital signs					
Temperature	85	26.0	213	74.0	< 0.001
Blood pressure	289	88.0	288	99.0	< 0.001
Pulse	111	34.0	273	93.5	< 0.001
Respirations	13	4.0	172	60.0	< 0.001
Composite	3	1.0	160	55.0	< 0.001
Fetal well being					
Fundal height^†^	167	98.0	199	100.0	0.030
Heart rate (BCF)^†^	167	98.0	194	97.5	0.914
Movement (after 20 weeks)^†^	80	47.0	197	99.0	< 0.001
Position (after 36 weeks)^‡^	82	95.4	89	98.0	0.367
Composite	121	37.0	259	89.0	< 0.001
Counseling					
Needed supplies are available	224	68.0	215	75.0	0.050
Counseling occurs in private room	304	92.1	288	99.0	< 0.001
Makes eye contact with woman	291	88.1	287	98.2	< 0.001
Speaks to woman in respectful manner	316	96.0	289	99.0	0.014
Uses words that woman can understand	294	89.0	285	98.0	< 0.001
Concrete response provided	78	24.0	199	68.0	< 0.001
Explains all medical procedures	44	13.3	269	93.4	< 0.001
Composite	7	2.2	149	51.0	< 0.001

^†^*N*= 171 for baseline and *N*= 199 for follow-up

^‡^*N*= 86 for baseline and *N*=91 for follow-up

The effect of MESH-QI on the danger sign assessment score was modified by district and type of ANC visit (*p*-value for interaction< 0.001, Table 3). No significant interaction was found between the effect of MESH-QI and FANC training (*p* = 0.436) and level of mentee’s education (*p* = 0.101). After controlling for level of mentee’s education and FANC training and clustering at nurse level, the MESH-QI intervention remained associated with significant improvement in the danger sign assessment score (Table 4). However, the effect of the MESH-QI intervention on the danger sign assessment score was different for each district and type of ANC visit: For Southern Kayonza District, the predicted increase in danger sign assessment score under MESH-QI was 6.28 (95% CI: 5.59, 6.98; *p* <  0.001) for non-first ANC visits, and 5.39 (4.62, 6.15; *p* <  0.001) for first ANC visits. For Kirehe District, the predicted increase in danger sign assessment score was 4.20 (95% CI: 3.59, 4.80; *p* <  0.001) for non-first ANC and 3.30 (95% CI: 2.80, 3.81; *p* <  0.001) for first ANC visits.

**Table 3:** Relationship between demographic characteristics and danger sign assessment score and mentoring period, stratified by demographics characteristics

	Bivariate analysis		*P*-value for interaction term
Predictors	Changes in ANC Assessment Score	95% CI	
District			< 0.001
Southern Kayonza			
Baseline	Ref.		
Post-MESH-QI	6.06	[5.43, 6.69]	
Kirehe			
Baseline			
Post-MESH-QI	3.88	[3.46, 4.30]	
FANC Training			0.436
Received FANC Training			
Baseline	Ref.		
Post-MESH-QI	4.75	[4.15, 5.35]	
Did not receive FANC training			
Baseline	Ref.		
Post-MESH-QI	4.47	[4.03, 4.91]	
Level of education			0.101
High education			
Baseline	Ref.		
Post-MESH-QI	5.90	[4.27, 7.54]	
Secondary education			
Baseline	Ref.		
Post-MESH-QI	4.50	[4.13, 4.87]	
ANC visit			< 0.001
First ANC visits			
Baseline	Ref.		
Post-MESH-QI	5.05	[4.53, 5.57]	
Other ANC visits			
Baseline	Ref.		
Post-MESH-QI	3.84	[3.38, 4.30]

**Table 4:** Changes in danger sign assessment score post-MESH-QI intervention^a^

	Changes in assessment score	95% CI
The effect of MESH-QI, Kirehe, non-first ANC	4.20	[3.59, 4.80]
The effect of MESH-QI, Kayonza, non-first ANC	6.28	[5.59, 6.98]
The effect of MESH-QI, Kirehe, first ANC	3.30	[2.80, 3.81]
The effect of MESH-QI, Kayonza, first ANC	5.39	[4.62, 6.15]

^a^Controlling for FANC training and level of mentee’s education

## Discussion

Although ANC represents an important opportunity to detect danger signs during pregnancy [26] and ensure appropriate management of pregnancy risks [53], there is a need of attention to quality of ANC delivery in resource-limited settings. This study’s findings demonstrate that MESH-QI model strengthens the quality of ANC as measured by improvement in the danger sign assessment score. The observed improvements persist even when controlling for FANC-training status and level of nurse-mentee’s education, and were greater for non-first ANC visits, both of which had lower danger sign assessment scores at baseline. The findings suggest MESH-QI as a promising intervention to improve components of quality of care in resource-limited settings facing staffing challenges including low levels of training and education. These results are consistent with the growing evidence highlighting the need for enhanced and effective supervision after didactic trainings [19].

Although overall danger sign assessments and most other assessment items were more likely to be completed under MESH-QI, some screening areas did not improve. For fetal position and heart rate, the completeness was high at baseline and stayed persistently high. For history assessment, even though there was a significant improvement during the MESH-QI intervention period, the levels of completeness under MESH-QI remained poor. We have several hypotheses that could explain this result. First, mentors may have emphasized strengthening danger sign assessments assuming that the woman’s history was already known from previous visits. Furthermore, nurse-mentees were residents of the health center catchment area, and it is possible that they had opportunities to interact with women outside of clinic and therefore deprioritized a systematic woman’s history assessment during ANC visit. The lack of essential tools to guide clinical supervision may have led to notable inconsistencies prior to MESH-QI intervention. The use of standardized checklists as part of MESH-QI intervention helped to assess and improve nurse-mentee’s competencies and address systems gaps.

This study has important limitations to consider in interpreting results. First, the pre-post design without a control means that we cannot make definitive conclusions about attribution. However, there were no other ANC-targeted quality improvement work in the two districts and no changes in national ANC strategy or other ANC-focused interventions during the study period other than periodic FANC training or increased nurse education, which we controlled for in the final analysis. Another limitation is that we relied on performance measurements collected during routine mentoring visits by mentors themselves, who may introduce bias in their observation of ANC assessments. Furthermore, a Hawthorne effect may have caused ANC nurses to perform better as a result of being observed resulting in overestimates of the overall effect of the MESH-QI intervention. However, mentors were trained in relationship building and other techniques as part of their orientation. We believe this reassured nurse-mentees so that they were able to provide their usual care without fear of judgment.

In the efforts to promote the universal health coverage, Rwanda successfully launched a community-based health insurance scheme, “Mutuelle” [54]. Local district officials incorporated *mutuelle* on the list of targets for district performance contracts locally known as “Imihigo” [55]. This study’s baseline data were collected during the evaluation of the district performance [56], a period marked by intensive efforts deployed by districts to accelerate the pace toward performance goals. This efforts may have increased *mutuelle* enrollments, leading to increased utilization of health center services. Furthermore, an increased workload may have caused an intra-clinic pressure with indirect effect on baseline findings. As such, nurses may have rushed to complete consultations with limited time to focus on recommended ANC practices.

Finally, we sought to assess the effect of the MESH-QI model on danger sign assessments and other ANC screenings. We assume that improving key ANC assessments has improved case management. Further studies are needed to assess the effect of the MESH-QI intervention on pregnancy outcomes. Future studies should also assess the impact of the MESH-QI on other aspects of the nurse-mentees including satisfaction, retention and perceived impact on their clinical competencies. Future studies should also assess the impact of the MESH-QI on other aspects of nurse-mentees’ experiences including satisfaction, retention and perceived impact on their clinical competencies. Moreover, we recommend exploring the experiences of pregnant women using ANC services and the impact of MESH-QI on these experiences. This information is crucial to understand their perceptions as well as improvements needed to better meet patient expectations.

While ANC is critical to strengthen maternal and newborn health outcomes, the failure of training and supervision to improve the quality of care suggests the need for evidence-based interventions to improve ANC quality in sub-Saharan Africa [57]. This study demonstrates the benefits of a mentorship intervention, MESH-QI, to improve the quality of ANC at rural health centers. As such, this constitutes an invaluable contribution to the WHO’s goal to have a world where “every pregnant woman and newborn receives quality care throughout the pregnancy, childbirth and the postnatal period” [58] and is consistent with their recommendation to promote health systems interventions that improve the utilization and quality of ANC [59].

## Conclusion

In resource-constrained settings where the application of clinical skills constitutes a major challenge, MESH-QI could be an effective model to improve the quality of ANC and increase the opportunities to early detect and manage pregnancy complications.

This study highlights the importance of post-training mentoring and quality improvement rather than relying solely on didactic trainings and traditional supervision. Further, updated guidelines and observation checklists are key for mentors or supervisors to have a systematic view of ANC and provide feedback. In order to sustain these improvements, efforts are underway to integrate the MESH-QI checklists and quality of care indicators into routine district supervision and health management information system.

## Abbreviations

DH: District hospital
FANC: Focused antenatal care
IMAI: Integrated Management of Adolescent and Adulthood Illness
IMCI: Integrated Management of Childhood Illness
MDGs: Millennium development goals
MESH-QI: Mentorship, enhanced supervision for healthcare and quality improvement
MoH: Ministry of Health
MoH: Ministry of Health
PIH: Partners In Health
